# Robotic Surgery in Gynecology: Balancing Clinical Benefit, Cost-Effectiveness, and Accessibility

**DOI:** 10.3390/jcm15103628

**Published:** 2026-05-09

**Authors:** Dario Colacurci, Giuseppe Bifulco, Mario Ascione, Ina Shehaj, Morva Tahmasbi Rad, Khayal Gasimli, Sven Becker

**Affiliations:** 1 Department of Obstetrics and Gynecology, Johann Wolfgang Goethe University, Theodor-Stern-Kai 7, Haus 15, 60590 Frankfurt, Germany; inashehaj@hotmail.com (I.S.); morva.tahmasbirad@kgu.de (M.T.R.); khayal.gasimli@kgu.de (K.G.); sven.becker@kgu.de (S.B.); 2 Department of Public Health, University Federico II, via Pansini 5, 80131 Naples, Italy; giuseppe.bifulco@unina.it (G.B.); mario.ascione@unina.it (M.A.)

**Keywords:** robotic-assisted surgery, gynecologic surgery, cost-effectiveness, health equity, minimally invasive surgery, healthcare policy

## Abstract

**Background:** Robotic-assisted surgery (RAS) has progressively expanded in gynecologic practice. Although its technical advantages are recognized, its economic sustainability and equitable accessibility remain debated. **Methods:** This clinical update provides a critical narrative review of current evidence on RAS in gynecology, integrating data on clinical outcomes, cost-effectiveness, diffusion patterns, and health equity across different healthcare settings. **Results:** In both benign and oncologic indications, RAS demonstrates consistent perioperative advantages over open surgery, including reduced blood loss, shorter hospital stay, and lower conversion rates. In routine cases, outcomes are largely comparable to conventional laparoscopy. However, robotic approaches appear particularly beneficial in complex scenarios, such as obesity, advanced malignancy, and technically demanding procedures. Economic evidence is heterogeneous. Short-term hospital-based studies report higher direct costs for RAS, especially in benign surgery. Conversely, cost–utility models in oncologic settings suggest that RAS may achieve acceptable cost-effectiveness when long-term outcomes, quality-adjusted life years, and institutional volume are considered. Accessibility remains strongly influenced by reimbursement policies, procedural volume, infrastructure, and workforce training. In the absence of structured reimbursement frameworks, robotic surgery may contribute to socioeconomic and geographic disparities. **Conclusions:** RAS represents an important component of modern gynecologic surgery, particularly in high-complexity and high-risk cases in which its technical advantages may translate into meaningful perioperative benefit. Its long-term sustainability depends on appropriate patient selection, institutional volume, reimbursement models, and health system organization. Future research incorporating long-term and societal economic perspectives is required to support balanced and equitable implementation.

## 1. Introduction

Over the past two decades, robotic-assisted surgery (RAS) has become an increasingly important tool of gynecologic surgical practice [1]. Its introduction has improved precision, visualization, and ergonomics [2] in minimally invasive surgery, and its use has progressively expanded worldwide across both benign [3] and oncologic indications. Recent evidence suggests that robotic-assisted surgery may offer perioperative advantages, particularly when compared with open surgery, including reduced blood loss [4], shorter learning curves [5], and lower conversion rates to laparotomy. These characteristics have supported the adoption of RAS in selected complex settings, including obesity, where technical and ergonomic benefits may help minimally invasive surgery and may be associated with earlier recovery and fewer readmissions [6,7]. However, overall superiority over advanced laparoscopic techniques has not yet been demonstrated, and recent literature is still heterogeneous. Indeed, various clinical trials show comparable outcomes between RAS and other surgical settings [1]. Nevertheless, hospitals and gynecology departments must consider a substantial financial investment when including a robotic-assisted theatre in their facility: acquisition cost, maintenance fees, disposable instruments, and training requirements are the principal charges for healthcare institutions [8,9]. Its cost-effectiveness is still a major concern, especially in low-resource settings and low-volume centers. Beyond economic considerations, access to robotic surgery is also influenced by healthcare system organization, reimbursement policies, and procedural volume, raising important concerns regarding health equity. Economic evaluations should consider procedural volume, long-term outcomes, surgeon expertise, and case complexity. Accordingly, the choice of surgical approach should be individualized according to patient characteristics, disease complexity, and institutional resources. Rather than asking whether RAS is superior to existing approaches, a more relevant question is posed: under which clinical, economic, and organizational conditions it provides meaningful added value. The aim of this clinical update is to provide a critical overview of current evidence on the cost-effectiveness and accessibility of robotic surgery in gynecology. By integrating clinical outcomes, economic evaluations, and real-world considerations across different healthcare settings, this review aims to support informed decision-making and promote sustainable, patient-centered surgical care.

## 2. Materials and Methods

This study is a narrative review aimed at providing a critical overview of the clinical outcomes, cost-effectiveness, and accessibility of robotic-assisted surgery in gynecology. Two independent authors performed a literature search in PubMed/MEDLINE and Scopus, covering studies published from database inception to March 2026. To ensure a balanced evaluation of the topic, the literature search was structured to capture evidence across two main domains: (1) clinical outcomes and (2) economic evaluations of robotic-assisted surgery. For clinical outcomes, the search strategy combined terms related to “robotic surgery”, “robotic-assisted surgery”, “gynecology”, “hysterectomy”, and “minimally invasive surgery”. For economic analyses, additional keywords such as “cost-effectiveness”, “cost analysis”, and “health economics” were included. Although a unified search framework was applied, keyword combinations were adapted according to the specific domain of interest to ensure appropriate coverage of both clinical and economic evidence. Study selection was based on relevance to the scope of the review. Priority was given to high-quality evidence, including systematic reviews, meta-analyses, randomized controlled trials, and large observational studies. Additional relevant publications were identified through manual screening of the reference lists of selected articles. Case reports, editorials, and studies not directly relevant to gynecologic robotic surgery were not prioritized. Given the narrative nature of this review, no formal risk-of-bias assessment tool was applied. However, study quality was critically evaluated during the selection process, regarding study design, sample size, methodological robustness, and consistency of findings. The inclusion of heterogeneous studies reflects the complexity of the topic: it was considered necessary to describe an integrated perspective across clinical, economic, and organizational domains. Evidence from different study designs was synthesized qualitatively and interpreted contextually, rather than quantitatively pooled.

## 3. Current Diffusion of Robotic Surgery

**Figure 1:**
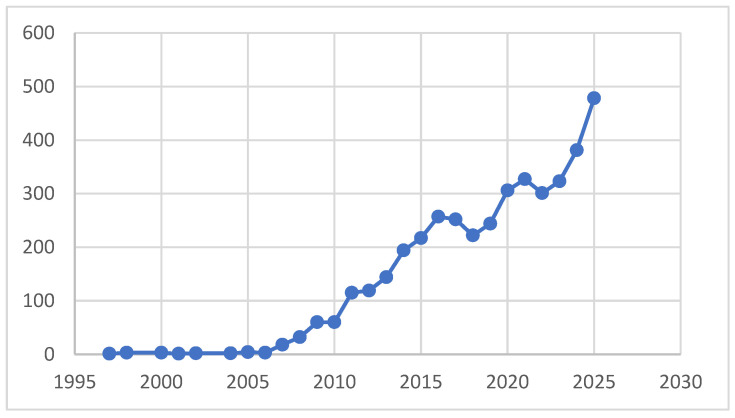
Temporal trend in PubMed publications on robotic surgery in gynecology (inception to March 2026). Data derived from a PubMed/MEDLINE search using the keywords “robotic surgery gynecology”.

Although initially limited to highly specialized centers, robotic platforms are now widely available in tertiary referral hospitals and, in some regions, in secondary-level ones. Technological innovation has driven this diffusion, together with increasing surgeon expertise, institutional investment, and patient preference for minimally invasive procedures. The progressive use of RAS is, indeed, mirrored in the constant growth of scientific publications. A PubMed search using the phrase “robotic surgery gynecology” shows an exponential increase in annual publications, from sporadic reports in the early 2000s to more than 300 annually from 2020 onward and approaching 500 publications in 2025. This trend reflects its expansion both in clinical use and in growing scientific interest in outcomes, safety, training, and economic implications. Figure 1 shows this increasing trend.

This bibliometric evolution is supported by recent evidence. Peng et al. [10] have shown three phases of research development in the health economics of robotic surgery: a slow-growing period, a developing phase, and a rapid expansion phase. Major contributions to scientific literature have come from high-income countries, particularly the United States, China, and several European nations. Gynecology represents one of the principal clinical fields within this research domain. Additionally, market analyses confirm bibliometric data evaluating the global expansion of robotic surgical systems. Surgical Robot Market 2025–2033 Report [11] estimated the global market at circa 4.3 billion USD in 2024. Moreover, the same projection has shown the market surpassing 9 billion USD by 2033, with an annual growth rate above 9%. Despite continuous market growth, RAS limited diffusion is due principally to high acquisition costs and regulatory barriers. Moreover, the lack of reimbursement mechanisms and insufficient training infrastructures increases the difficulty of RAS spread in these settings, raising global differences in access to advanced surgical technologies. However, in the Middle East and Africa, the Surgical Robot Market 2025–2033 Report [11] has shown a gradual expansion of robotic surgery: increasing oncologic burden and demand for specialized care are driving the shift. In these regions, RAS utilization is often supported by alternative financing models, such as pay-per-use systems. While such arrangements improve short-term management, large-scale availability with long-term sustainability depends on institutional resources, workforce training, and healthcare financing structures. Historically, early European adoption schemes helped illustrate the role of economic and demographic factors. Vaessen [12] showed that most da Vinci systems in 2010 were concentrated in high purchasing power countries and/or in regions with high population density. These historical patterns demonstrate how healthcare policies and financial sustainability have shaped diffusion. In high-income countries, robotic surgery is now an integral component of gynecologic care [13], particularly in oncologic and complex pelvic procedures. High procedural volumes and structured training programs have facilitated integration into routine practice. Interdepartmental collaboration has been crucial for its widespread adoption [14]. Robotic systems are often shared among multiple specialties, such as gynecology, urology, general surgery, and colorectal surgery, which increases RAS utilization [15]. This multidisciplinary approach increases case volume, optimizes operating room efficiency, and distributes fixed costs across departments. Although robotic platforms require substantial initial investment, these fixed costs can be progressively amortized with increasing procedural volume. Higher case numbers reduce per-procedure costs through improved equipment utilization, optimized workflows, and reduced idle time. Consequently, high-volume centers, where multidisciplinary surgeons work together, reach economic balance more efficiently, compared with low-volume institutions where elevated costs and limited sustainability are still present. Surgeon training and learning curves represent additional determinants of adoption. As occurred during the early adoption of conventional laparoscopy, improved technical proficiency is strictly associated with standardized certification programs [16,17], simulation platforms [18], and mentorship networks; indeed, they contribute to improve patient safety, supporting technology diffusion. At the same time, the demand for robotic platforms increases market competition, which may facilitate broader access by reducing acquisition and maintenance costs, although long-term performance data remain limited. These structural, organizational, and economic factors provide the framework within which cost-effectiveness analyses must be interpreted. While RAS is widely established in high-resource settings, significant barriers are still present in low-income realities. Cost-effectiveness data interpretation is strictly dependent on understanding these contextual factors.

## 4. Clinical Outcomes

### 4.1. Benign Gynecologic Surgery

Several systematic reviews and meta-analyses have evaluated the clinical outcomes of robotic-assisted surgery in benign gynecologic procedures, particularly hysterectomy and pelvic floor reconstruction. A large meta-analysis by Louis Lenfant et al. [19] compared robotic-assisted hysterectomy with laparoscopic, vaginal, and open approaches for benign indications, including over one million patients. In the meta-analysis by Lenfant et al. [19], robotic hysterectomy was associated with less blood loss, shorter hospital stays, and fewer complications than open surgery, while compared with conventional laparoscopy the main consistent advantage was a slightly shorter hospital stay (WMD −0.14 days), with otherwise broadly comparable perioperative outcomes. No significant differences were observed in overall complication, mortality, or reoperation rates. Similarly, in urogynaecologic surgery, a systematic review by Maribel De Gouveia De Sa et al. [20] evaluated minimally invasive versus open sacrocolpopexy for apical prolapse. Minimally invasive techniques, including robotic surgery, showed similar success and recurrence rates compared with open surgery. Although robotic-assisted surgery showed longer operative times, it reduced transfusion rates, length of stay, and estimated blood loss. Recent ESGE good practice recommendations on abdominal myomectomy [21] further support an individualized approach to minimally invasive surgery in benign gynecology. In these recommendations, robotic-assisted myomectomy is included within the laparoscopic approach and is considered a potentially valuable option in technically demanding cases, particularly because myomectomy is a suture-intensive procedure. At the same time, the choice between laparoscopic, robotic-assisted, and open surgery should be based on fibroid characteristics, patient factors, and, importantly, the expertise of the surgeon and surgical team. Endometriosis represents another relevant benign indication for minimally invasive surgery, particularly in complex cases of deep infiltrating disease. A recent meta-analysis [22] that included 14 studies and 2709 patients showed no significant differences between RAS and conventional laparoscopic surgery in terms of intraoperative complications (RR 1.64, 95% CI 0.55–4.86), postoperative complications (RR 0.95, 95% CI 0.78–1.17), conversion rates (RR 1.26, 95% CI 0.33–4.85), and estimated blood loss (SMD 0.03, 95% CI −0.08 to 0.14). However, RAS was associated with longer operative time (SMD 0.54, 95% CI 0.25–0.84; *p* < 0.0001) and slightly longer hospital stay (SMD 0.14, 95% CI 0.02–0.26; *p* = 0.020). Although outcomes are comparable, RAS may offer technical advantages in complex anatomical settings due to enhanced visualization, instrument articulation, and ergonomics.

### 4.2. Gynecologic Oncology

In gynecologic oncology, robotic-assisted surgery has been extensively investigated in endometrial, ovarian, and cervical cancer. A meta-analysis by Weimin Xie et al. [23] comparing robotic-assisted and conventional laparoscopic surgery for endometrial cancer demonstrated significantly lower blood loss, reduced conversion rates, and shorter hospital stay in the robotic group, while showing comparable rates of intraoperative injuries, transfusions, and lymph node biopsies. Specifically, Xie et al. reported lower estimated blood loss (WMD −77.65 mL), lower conversion to laparotomy (OR 0.29), and shorter hospital stay (WMD −0.48 days) with robotic-assisted surgery compared with conventional laparoscopy, while operative time, transfusion rate, and total lymph nodes harvested were comparable. More recently, the multicenter COMPARE Study [24], a large multispecialty analysis including different oncologic procedures, evaluated perioperative outcomes across seven oncologic procedures, including hysterectomy for gynecologic malignancies, although its findings should be interpreted with caution when applied to gynecologic surgery due to the absence of procedure-specific stratification. This large meta-analysis reported that robotic surgery was associated with significantly lower conversion rates, reduced blood transfusion requirements, shorter length of stay, fewer postoperative complications, and lower 30-day mortality compared with both laparoscopic and open approaches, despite longer operative times. In contrast, current ESGO–ESTRO–ESP guidelines for endometrial carcinoma [25] identify minimally invasive surgery as the preferred surgical approach for stages I–II disease, including high-risk patients, when performed in appropriate settings and by experienced teams. Within this framework, robotic-assisted surgery can be interpreted as a technological extension of minimally invasive surgery, whose advantages may be particularly relevant in selected complex cases. Instead, in ovarian cancer, a systematic review by Akira Yokoi et al. [26] compared minimally invasive surgery, including robotic techniques, with open laparotomy. The authors found no significant differences in overall or progression-free survival between approaches, while minimally invasive surgery was associated with shorter hospital stay and reduced blood loss. However, advanced-stage disease requires careful consideration, and the authors underscore the importance of careful patient selection in this subgroup. In ovarian cancer, however, current ESGO–ESMO–ESP consensus recommendations [27] emphasize that oncologic outcomes are primarily driven by the achievement of complete cytoreduction, rather than by the surgical platform itself. Therefore, the potential role of robotic-assisted surgery in ovarian cancer should be interpreted with caution and limited to highly selected settings in which complete resection is not compromised. In early-stage disease, minimally invasive approaches may be considered for staging procedures in carefully selected patients. In contrast, in advanced ovarian cancer, where extensive cytoreductive surgery is required, the role of minimally invasive and robotic approaches remains limited and should be restricted to highly selected cases. Moreover, for early-stage cervical cancer, recent meta-analyses [28] have reported comparable short-term oncologic outcomes between robotic and laparoscopic approaches, with robotic surgery demonstrating lower conversion rates and improved perioperative profiles in selected patients. A pivotal contribution to this field is represented by the LACC trial [29], a multicenter randomized study comparing minimally invasive and open radical hysterectomy in early-stage cervical cancer. The trial demonstrated significantly lower disease-free and overall survival in the minimally invasive group (4.5-year disease-free survival 86.0% vs. 96.5%; hazard ratio for recurrence or death 3.74), raising concerns about the oncologic safety of minimally invasive approaches in this setting. However, only a minority of patients in the minimally invasive arm underwent robotic-assisted surgery, and no direct comparison between laparoscopic and robotic techniques was performed. This interpretation is consistent with current ESGO resource-stratified recommendations for cervical cancer [30], which emphasize centralization of care, multidisciplinary planning, and adaptation of surgical strategies according to disease setting, expertise, and available resources. In particular, for procedures requiring radical parametrectomy, laparotomy remains the standard approach, supporting a cautious and context-dependent interpretation of minimally invasive approaches in cervical cancer. While robotic-assisted surgery may offer perioperative advantages in selected settings, its oncologic role cannot be generalized across all gynecologic malignancies.

### 4.3. Special Populations and Technical Advantages

Beyond standard indications and comparisons with other surgical techniques, robotic-assisted surgery may offer potential advantages in complex surgical scenarios and selected populations, although the available evidence remains heterogeneous. Obese patients have increased risks of perioperative complications [31,32] as they more frequently present with comorbidities that increase anesthesiologic risk [33]. Moreover, scientific literature shows that this population has an overall increased conversion rate [34]. Meta-analyses focused on obese patients suggest that robotic-assisted surgery may be associated with lower conversion rates and reduced blood loss compared with conventional laparoscopy; however, quantitative thresholds and subgroup-specific effects remain insufficiently defined [35]. In obese patients with endometrial cancer, Cusimano et al. [35] reported pooled conversion rates of 6.5% for laparoscopy and 5.5% for robotic hysterectomy in patients with BMI ≥ 30 kg/m^2^, and 7.0% versus 3.8%, respectively, in patients with BMI ≥ 40 kg/m^2^. Overall complication rates were low and broadly similar, but intolerance of the Trendelenburg position accounted for 31% of laparoscopic conversions versus 6% of robotic conversions. Similarly, in other technically demanding settings, such as extensive adhesions, large uteri, previous abdominal surgery, or advanced malignancy, robotic surgery has also been associated with lower conversion rates [36,37]. Reduced conversion rates are clinically relevant, as conversion is associated with increased morbidity, prolonged hospitalization, and higher healthcare costs. Nevertheless, increased precision and stability of robotic instruments may partially overcome surgeon experience limits, improving procedural safety in high-complexity settings. Taken together, these findings suggest that the potential clinical value of robotic surgery may become more evident in selected high-complexity settings, although further quantitative evidence is required to better define its impact across different patient subgroups.

### 4.4. Summary of Clinical Outcomes

Across benign and malignant gynecologic indications, robotic-assisted surgery demonstrates:

- Comparable effectiveness to conventional laparoscopy in routine cases;
- Superior perioperative outcomes compared with open surgery;
- Potentially lower conversion rates in complex and high-risk patients;
- Reduced blood loss and length of hospital stay;
- Similar rates of major complications and mortality.

While operative times are generally longer with robotic approaches, these differences do not appear to compromise patient safety or clinical effectiveness. Instead, the technical advantages of robotic platforms seem to enhance surgical performance in selected contexts, supporting their role in modern gynecologic practice. Taken together, the available evidence suggests that the added value of robotic-assisted surgery does not lie in outperforming laparoscopy in routine cases, but in providing greater procedural stability and lower conversion rates in technically challenging scenarios. Table 1 summarizes the main clinical studies identified through the structured literature search described in the Methods section.

## 5. Cost Analysis

Economic outcomes reported in the literature are heterogeneous in nature and derive from different analytical approaches. Cost–utility analyses typically provide model-based, long-term estimates expressed as quality-adjusted life years (QALYs) and incremental cost-effectiveness ratios (ICERs). QALYs are a measure that combines both survival and quality of life, while ICERs express the additional cost required to gain one additional QALY when comparing different surgical techniques. These analyses generally adopt a long-term or lifetime perspective and are interpreted in relation to country-specific willingness-to-pay thresholds. In contrast, studies reporting direct costs (e.g., hospitalization or procedural costs) reflect short-term, hospital-based analyses and do not account for long-term outcomes or quality-of-life adjustments. For this reason, these economic measures are not directly comparable and should be interpreted within their respective methodological frameworks. Cost data are not directly comparable across countries due to differences in currency, reimbursement systems, and healthcare organization; therefore, results should be interpreted within their specific national and methodological context rather than as universally generalizable. While robotic surgery has been widely adopted based on its technical and clinical advantages, the available economic evidence remains heterogeneous and often context-specific. Most short-term, hospital-based studies consistently report higher direct costs associated with robotic procedures. In the French cost-effectiveness analysis comparing robotic-assisted myomectomy with open surgery, Boyer de Latour et al. [38] observed an additional mean cost of €3555 per patient for robotic surgery, mainly driven by investment, maintenance, consumables, and operating room occupancy time. Despite shorter hospital stays (2.98 vs. 4.34 days) and lower readmission rates (0% vs. 1.21%), robotic surgery did not significantly reduce major perioperative complications. Consequently, the incremental cost-effectiveness ratio reached €155,241 per complication avoided, indicating an unfavorable economic profile in the setting of benign and complex myomectomies. This study illustrates that modest clinical advantages in recovery parameters may be insufficient to compensate for the high fixed costs of robotic platforms in non-oncological settings. On the other hand, evidence suggests that economic performance of robotic surgery may improve when survival and quality-of-life outcomes are analyzed. In a Chinese model-based cost–utility analysis with a long-term perspective [39], robotic surgery was associated with a gain of 4.84 quality-adjusted life years, despite higher initial costs exceeding 1,000,000 RMB. The incremental cost-effectiveness ratio remained below the national willingness-to-pay threshold, especially in high-volume centers performing over 138 procedures annually. This study highlights the limits of short-term cost analyses: the true value of robotic surgery in oncological contexts is long-term disease control and postoperative quality of life. This divergence between short-term cost analyses and long-term cost–utility findings reflects a fundamental issue: the economic value of robotic surgery is not driven by immediate perioperative gains alone, but by its potential impact on downstream outcomes, including recovery trajectory, quality of life, and continuity of oncologic treatment. It should be noted that these values represent model-derived, incremental estimates evaluated over a long-term time horizon, rather than direct per-patient or short-term hospital costs. The importance of surgical volume is crucial in economic balance and sustainability, as shown in several studies [40,41]. In the French analysis [38], cost-volume modeling showed that even maximal robot utilization could not fully offset the cost difference with open surgery, although the gap narrowed substantially. Likewise, the Chinese model [39] showed that cost-effectiveness was closely linked to institutional case volume. These results suggest that concentrating robotic procedures in high-volume centers may be required to ensure acceptable economic outcomes. System-level evidence further confirms the persistent cost premium of robotic surgery. The recent systematic review and meta-analysis conducted in South Korea [8], including 24 studies published between 2007 and 2025, stated that robotic-assisted surgery was associated with a mean increase of $3279 in total hospitalization costs and $3589 in operating costs compared with laparoscopic surgery. Moreover, patients undergoing RAS had an additional $5701 in out-of-pocket costs, while government reimbursement was substantially lower. These findings underscore a significant financial burden on patients, raising concerns about equitable access, particularly in healthcare systems where robotic surgery is not publicly funded. Additionally, this Korean review [8] highlighted a critical methodological gap: none of the included studies incorporated formal cost–utility analyses using quality-adjusted life years or long-term modeling approaches. Most investigations were limited to short-term hospitalization costs and single-center observational designs, resulting in very high heterogeneity (I^2^ up to 100%). The lack of comprehensive economic evaluations limits the ability to draw solid conclusions regarding the long-term value of robotic surgery. Moreover, Eoh et al. [42] showed in a nationwide database analysis of hysterectomy procedures that although initial hospitalization costs were higher for robotic surgery, post-discharge emergency room visits and outpatient costs were lower compared with open surgery. This suggests potential downstream savings that are not captured in short-term hospital-based evaluations. Taken together, these studies suggest that the economic value of robotic surgery is strongly disease-specific, with limited short-term value in benign surgery but potentially more favorable profiles in selected oncologic settings when long-term outcomes are considered [38,39]. Economic conclusions also vary according to analytical perspective, time horizon, and modeling assumptions, while PROs and utility-based outcomes remain underrepresented in the literature [8,39]. Furthermore, learning-curve effects appear to play a non-negligible role. Several studies reported longer operative times in robotic groups, particularly during early adoption phases [8,38]. Prolonged operating room occupancy substantially increases procedural costs and may bias early economic evaluations against robotic platforms. As surgeon experience increases and workflows become optimized, these disadvantages may progressively diminish, potentially altering long-term cost profiles. The present findings also highlight important policy implications. The Korean experience [8,42] shows that, without public reimbursement, robotic surgery may widen socioeconomic disparities by limiting access to patients with greater financial means. Similar challenges may arise in other healthcare systems. Here, reimbursement models have not kept pace with technological advances. So, solid cost-effectiveness evidence is crucial to inform sustainable policy decisions with equity. In selected instances, evidence from multispecialty settings was included to provide a broader system-level perspective, particularly in health economic evaluations where gynecology-specific data are limited. However, these data cannot be directly extrapolated to gynecologic surgery, as clinical pathways, resource utilization, and outcomes may differ substantially across surgical specialties. Therefore, such findings should be interpreted with caution. Gynecology-specific evidence was prioritized whenever available.

Several limitations of the current body of evidence must be acknowledged. Most available studies are retrospective, single-center, and characterized by limited sample sizes [8]. Long-term follow-up is rarely available, and comprehensive modeling studies remain scarce [39]. Moreover, heterogeneity in surgical techniques, platforms, institutional organization, and reimbursement systems further complicates cross-study comparisons. Additionally, it should be acknowledged that cost data are derived from studies conducted over different time periods and may not fully reflect current costs, which are subject to change due to technological evolution, market competition, and evolving reimbursement policies. In light of these considerations, future research should focus on large-scale, multicenter economic analyses that incorporate quality-adjusted life years, longer time horizons, and a societal perspective. This would be important to clarify if the clinical benefits of robotic surgery are associated with sustainable economic value. Until then, robotic-assisted gynecologic surgery should be implemented selectively, with careful attention to oncologic indications, institutional case volume, and the specific characteristics of the healthcare system. Table 2 summarizes the key economic studies identified through the literature search described in the Methods section.

## 6. Accessibility and Health Equity

Robotic-assisted surgery diffusion in gynecology has not mirrored homogeneous accessibility across healthcare systems. As previously discussed, accessibility is influenced not only by financial investment but also by reimbursement policies, procedural volume, training infrastructure, and health system organization. Evidence from high-income countries has shown how reimbursement systems directly affect access to robotic-assisted surgery. In South Korea, where robotic procedures are not universally reimbursed, RAS patients have substantially higher out-of-pocket expenses compared with laparoscopic surgery, although comparable oncologic outcomes [8]. These financial differences may limit access for patients with lower economic resources, exacerbating socioeconomic disparities. When innovative technologies are introduced without corresponding reimbursement adaptation, equity issues become central to policy discussion. Without parallel adaptation of reimbursement frameworks and training infrastructure, the diffusion of robotic surgery risks reinforcing a two-tier healthcare model, in which access to advanced surgical technologies becomes increasingly dependent on geographic location and financial capacity. National experiences help to illustrate this variability. In Poland, robotic unit number has increased rapidly: over 10,000 procedures are performed annually through 80 robotic units distributed across the country [43]. Although this expansion, nearly half of robotic surgeons do less than 40 cases per year, suggesting significant procedural volume fragmentation. Regional disparities in case distribution were also observed: significant RAS differences are observed across administrative provinces [43]. These data suggest that technological expansion alone does not guarantee equity or efficient implementation. Low procedural volume per surgeon may compromise technical competence; moreover, it may delay the learning curve and limit the potential clinical and economic benefits of robotic platforms. Procedural volume fragmentation is particularly relevant in gynecologic oncology. High-volume centers are more likely to obtain optimized workflows, reduced operative times, and improved cost amortization [38,44]. However, RAS-specialized centers may generate geographic inequities, forcing patients to travel long distances to access minimally invasive oncologic care. Balancing procedural workflows and regional accessibility is still a complex structural challenge. In contrast, RAS integration in low- and middle-income countries (LMICs) is characterized by additional barriers. A major financial obstacle is implementation fees exceeding one million USD per robotic unit, and per-procedure fees ranging from 3000 to 5000 USD [45,46]. Moreover, infrastructural limitations, lack of trained surgeons, lack of standardized curricula, and inadequate digital connectivity limit feasibility. Although robotic surgery may reduce surgical site infections [47], hospital stay, and overcrowded surgical waiting lists [19], its diffusion may exacerbate inequalities if access remains limited to wealthier urban centers. Training disparities represent another important limit of equity. RAS utilization without structured national curricula or standardized registries may result in inadequate skill acquisition and heterogeneous clinical outcomes [43]. Quality assurance goes with technological innovation, as suggested by countries in which standardized robotic curricula and centralized outcome monitoring were introduced immediately. Indeed, it becomes difficult to assess if RAS integration truly improves oncologic and perioperative outcomes without coordinated evaluation systems. Additionally, accessibility should not follow indiscriminate diffusion. In many routine benign gynecologic procedures, traditional laparoscopy is still clinically equivalent and more economically sustainable [44]. Adequate care requires careful patient selection; indeed, robotic approaches should be prioritized for cases where technical advantages are measurable benefits, such as complex oncologic resections, obese patients, and high-risk surgical scenarios. Finally, RAS integration in gynecologic surgery depends on a balance between reimbursement models, institutional case volume, structured training pathways, outcome monitoring systems, and national health policy frameworks. Sustainable implementation requires coordinated governance, evidence-based patient selection, and continuous evaluation to permit equitable access to high-quality surgical care.

## 7. Comparison with Alternative Surgical Approaches

Robotic-assisted surgery should not be considered as a replacement for traditional surgical procedures, as the use of RAS depends on the clinical complexity, the surgeon’s expertise, and the healthcare system. The advantages of RAS are consistent and significant compared with open surgery. As previously shown, for benign and malignant gynecologic procedures, robotic procedures are associated with less blood loss, shorter hospital stay, lower conversion rates, and lower perioperative complications [19,31,35,36]. Oncologic outcomes may be comparable in selected settings, although this remains strongly dependent on tumor type, stage, and surgical objective. In this context, robotic procedures can be considered an extension of minimally invasive procedures, with advantages over laparotomy, mainly regarding perioperative results, especially for complex procedures and high-risk patients. On the other hand, the comparison with traditional laparoscopic procedures is less evident, with RAS showing similar results for routine benign and oncologic procedures, with no advantages regarding major clinical outcomes, with longer operating times and increased costs [1]. However, robotic systems could be considered with technical advantages especially for difficult cases, such as severe obesity [35], malignancy [28,31], extensive adhesions, and anatomical complexity [35,36,37]. In these contexts, lower conversion rates and improved ergonomics result in significant benefits, in particular when surgeries are done in high-volume centers with experienced teams. From an economic point of view, traditional laparoscopy is still more cost-efficient in many standard indications [44]. However, no single approach is universally superior; the optimal choice depends on patient characteristics, disease complexity, surgeon expertise, and health system resources, as it reflects a balance between assistance, complexity, and sustainability. RAS can be a valuable aid in high-volume centers to perform minimally invasive surgery in the treatment of complex gynecologic oncologic diseases, while in simpler situations or in low-volume institutions, traditional laparoscopic techniques are still the most rational and sustainable approach. This evaluation is coherent with current international guidelines across gynecologic diseases [21,25,27,30]. In particular, in benign gynecology, surgical planning should be individualized according to disease characteristics, patient preferences, and surgical expertise. In endometrial cancer, minimally invasive surgery is currently the preferred surgical approach in appropriate candidates. In cervical cancer, minimally invasive radical surgery requires caution, particularly in light of evidence and current guideline recommendations. In ovarian cancer, the achievement of complete cytoreduction is the main determinant of surgical value. Taken together, these recommendations support the view that robotic-assisted surgery should be considered a complementary and selective tool rather than a universally superior alternative.

## 8. Future Perspectives

The future of robotic-assisted surgery in gynecology will be defined by technological evolution and market competition. Although early adoption was characterized by high costs and limited competition, the distribution of new robotic platforms will lead to cost reduction and improved accessibility. Such developments could modify the economic balance between robotic and traditional minimally invasive techniques. Integration with artificial intelligence, diagnostic imaging systems, and intraoperative data assistance represents another interesting direction. Interestingly, intraoperative data analysis and augmented reality may help surgeons in terms of precision, potentially shortening the learning curves. However, these innovations must be validated in clinical and economic outcomes. Additionally, standardization of training programs will play a central role in future sustainability. Structured curricula with simulation-based certification programs may reduce variability in surgeon performance; centralized registries collecting long-term oncologic, perioperative, and patients’ outcomes are essential to have high-quality evidence. Moreover, economic research must also evolve. Future cost-effectiveness analyses should incorporate long-term data and quality-adjusted life years, including return to work and productivity metrics. Multicenter prospective studies are needed to evaluate in which indications robotic surgery is preferred to traditional laparoscopy. Particular attention should be given to high-complexity oncologic cases, obesity, and high-complex pelvic procedures, where robotic advantages may be most pronounced. Finally, health systems will need to balance centralization and access with equity. High-volume centers may compare economic and clinical performance; moreover, regional referral networks should guarantee that technological concentration does not translate into geographic or socioeconomic disparities. Sustainable RAS will depend on coordinated governance, improved reimbursement models, and continuous reassessment of value. In the coming years, robotic-assisted gynecologic surgery will not replace traditional laparoscopy. Instead, it may progressively redefine the limits of minimally invasive surgery in selected clinical contexts. Its long-term role will depend not only on technological innovation, but on the ability of healthcare systems to balance clinical benefit, economic sustainability, and equitable access. This review has some limitations, including the narrative nature of the synthesis, the heterogeneity of included studies, and the variability in healthcare systems, reimbursement models, and surgical expertise across countries. An additional limitation regards the inclusion of evidence derived from multispecialty studies, particularly in economic and system-level analyses. Although this approach was adopted to address gaps in gynecology-specific literature, it may reduce the direct applicability of findings to gynecologic practice. Future research should prioritize specialty-specific analyses to improve the precision and clinical relevance of economic and organizational evaluations.

**Highlights**

- Robotic surgery improves perioperative outcomes vs. open gynecologic surgery;
- Comparable outcomes to laparoscopy in routine benign and oncologic cases;
- Greatest clinical benefit observed in complex and high-risk scenarios;
- Higher upfront costs, with cost-effectiveness dependent on volume and context;
- Long-term value may emerge in oncologic care through QALY-based analyses;
- Access disparities persist, influenced by reimbursement and health system factors.

## 9. Conclusions

Robotic-assisted surgery has established itself as a valuable component of modern gynecologic practice, particularly in complex and high-risk clinical scenarios. However, its role is not defined by universal superiority, but by conditional benefit. The current body of evidence indicates that while robotic surgery improves perioperative outcomes compared to open techniques, it offers limited additional advantage over conventional laparoscopy in routine cases, at a substantially higher cost. Its greatest clinical contribution appears in technically demanding settings, where reduced conversion rates and enhanced surgical control may justify its use. From an economic perspective, the value of RAS is highly context-sensitive and closely linked to institutional volume, learning curve effects, and the analytical framework applied. Importantly, the lack of standardized reimbursement models and uneven global distribution raise critical concerns regarding equitable access. The future of robotic-assisted gynecologic surgery will depend not only on technological innovation, but on the ability of healthcare systems to integrate clinical benefit with economic sustainability and social equity. A more selective, evidence-driven implementation strategy, supported by long-term outcomes and real-world data, will be essential to ensure that innovation translates into meaningful and accessible patient care.

## Figures and Tables

**Table 1:** Summary of clinical outcomes of evaluated studies.

Study/Year	Field	Population	Comparison	Main Outcomes	Conversion Rate	Main Conclusions
Lenfant 2022 [19]	Benign	Benign hysterectomy (>1M pts)	RAS vs. laparoscopy vs. Open vs. Vaginal	EBL, LOS, complications, mortality	Lower than open surgery	Comparable to laparoscopy; better perioperative outcomes vs. open
Gouveia De Sa 2015 [20]	Benign	Apical prolapse	MIS (incl. robotic) vs. Open	Recurrence, EBL, LOS, OT	Not reported	Similar success; ↓ EBL/LOS; ↑ OT
Pavone 2024 [22]	Benign	Deep endometriosis	RAS vs. laparoscopy	Complications, EBL, LOS, OT	No significant difference	Non-inferior to laparoscopy; longer OT and LOS; technical advantages in complex cases
Xie 2016 [23]	Oncology	Endometrial cancer	RAS vs. laparoscopy	EBL, LOS, complications	Lower	↓ EBL, ↓ LOS; similar safety
Ricciardi 2024 (COMPARE) [24]	Oncology	Multispecialty cancers	RAS vs. laparoscopy/VATS vs. Open	Complications, LOS, mortality	Markedly lower	↓ complications and mortality
Yokoi 2024 [26]	Oncology	Ovarian cancer	MIS (incl. robotic) vs. Open	OS, PFS, EBL, LOS	Lower	Comparable survival; better perioperative profile
Marchand 2023 [28]	Oncology	Cervical cancer	RAS vs. laparoscopy/Open	Complications, LOS, conversions	Lower	↓ LOS
Ramirez 2018 (LACC) [29]	Oncology	Cervical cancer	MIS (laparoscopy + RAS) vs. Open	DFS, OS, recurrence	3.5% (laparoscopy only)	Lower DFS (86.0% vs. 96.5%) and OS with MIS; limited applicability to robotic
Cusimano 2024 [35]	High-risk	Obese patients	RAS vs. laparoscopy	EBL, complications, LOS	Markedly lower	Lower conversion in obese patients

Notes: RAS, robotic-assisted surgery; EBL, estimated blood loss; LOS, length of stay; MIS, minimally invasive surgery; OT, operating time; OS, overall survival; PFS, progression-free survival. The arrow ↓ indicate a decreasing value.The arrow ↑ indicate an increasing value.

**Table 2:** Summary of cost-effectiveness and economic evidence on robotic-assisted surgery in gynecology.

Study	Setting	Procedure	Design	Main Outcome	Key Results	Main Message
Chen [39]	China	Cervical cancer	Model-based	ICER/QALY	ICER: 213,054 RMB/QALY (below WTP)	Long-term cost-effectiveness in oncology
Boyer De Latour [38]	France	Myomectomy	Retrospective	Cost/complication	ICER: €155,241	Limited value in short-term benign surgery
Hong [8]	Korea	Multispecialty	Meta-analysis	Hospital costs	+$3279 vs. laparoscopy	Higher upfront costs
Eoh [42]	Korea	Hysterectomy	Database	Post-discharge cost	Lower ER/OP costs	Possible downstream savings

Notes: ICER, incremental cost-effectiveness ratio; QALY, quality-adjusted life year; WTP, willingness-to-pay threshold; ER, emergency room; OP, outpatient care; RAS, robotic-assisted surgery.

## Data Availability

The data supporting the findings of this study are available from the corresponding author upon reasonable request.

## Abbreviations

The following abbreviations are used in this manuscript:

BMI	Body Mass Index
EBL	Estimated Blood Loss
ER	Emergency Room
ICER	Incremental Cost-Effectiveness Ratio
LMIC	Low- and Middle-Income Country
LOS	Length of Stay
MIS	Minimally Invasive Surgery
OP	Outpatient
OS	Overall Survival
OT	Operating Time
PFS	Progression-Free Survival
QALY	Quality-Adjusted Life Year
RAS	Robotic-Assisted Surgery
WTP	Willingness-to-Pay
